# Phosphoserine enhanced Cu-doped bioactive glass dynamic dual-network hydrogel for craniofacial bone defect repair

**DOI:** 10.1093/rb/rbad054

**Published:** 2023-05-17

**Authors:** Yuwei Liu, Gang Wang, Huitong Luo, Bangjiao Zhao, Muheng Liao, Qiyuan Dai, Maocai Li, Qingtao Li, Xiaodong Cao

**Affiliations:** School of Materials Science and Engineering, South China University of Technology, Guangzhou 510641, P.R. China; National Engineering Research Centre for Tissue Restoration and Reconstruction, Guangzhou 510006, P.R. China; School of Materials Science and Engineering, South China University of Technology, Guangzhou 510641, P.R. China; National Engineering Research Centre for Tissue Restoration and Reconstruction, Guangzhou 510006, P.R. China; School of Materials Science and Engineering, South China University of Technology, Guangzhou 510641, P.R. China; National Engineering Research Centre for Tissue Restoration and Reconstruction, Guangzhou 510006, P.R. China; School of Materials Science and Engineering, South China University of Technology, Guangzhou 510641, P.R. China; National Engineering Research Centre for Tissue Restoration and Reconstruction, Guangzhou 510006, P.R. China; School of Materials Science and Engineering, South China University of Technology, Guangzhou 510641, P.R. China; National Engineering Research Centre for Tissue Restoration and Reconstruction, Guangzhou 510006, P.R. China; School of Materials Science and Engineering, South China University of Technology, Guangzhou 510641, P.R. China; National Engineering Research Centre for Tissue Restoration and Reconstruction, Guangzhou 510006, P.R. China; School of Materials Science and Engineering, South China University of Technology, Guangzhou 510641, P.R. China; National Engineering Research Centre for Tissue Restoration and Reconstruction, Guangzhou 510006, P.R. China; School of Medicine, South China University of Technology, Guangzhou 510006, P.R. China; School of Materials Science and Engineering, South China University of Technology, Guangzhou 510641, P.R. China; National Engineering Research Centre for Tissue Restoration and Reconstruction, Guangzhou 510006, P.R. China; Key Laboratory of Biomedical Engineering of Guangdong Province, South China University of Technology, Guangzhou 510006, P.R. China; Key Laboratory of Biomedical Materials and Engineering of the Ministry of Education, South China University of Technology, Guangzhou 510006, P.R. China; Zhongshan Institute of Modern Industrial Technology of SCUT, Zhongshan, Guangdong 528437, P.R. China

**Keywords:** Cu-doped bioactive glass, phosphoserine, dynamic hydrogel, craniofacial bone defect

## Abstract

Flexible hydrogels containing various osteogenic inorganic constituents, which can accommodate complicated shape variations, are considered as ideal grafts for craniofacial bone defect reconstruction. However, in most hybrid hydrogels, poor interaction between the polymer network and particles has detrimental effects on hydrogel rheological and structural properties, clinical manipulation and repair efficacy. In this article, we designed and prepared a series of hyaluronic acid composite hydrogel containing Cu-doped bioactive glass (CuBG) and phosphoserine (PS), in which hyaluronic acid was modified by methacrylate groups and phenylboronic acid groups to form a double crosslinked network. PS acted as an interaction bridge of CuBG particles and HAMA-PBA network to improve the mechanical properties of the composite hydrogels. The CuBG/PS hydrogels exhibited suitable rheological properties (injectable, self-healing, shape-adaptable), bone tissue integrating ability and anti-bacterial property. Meanwhile, we found that CuBG and PS have synergistic effect on improving osteogenic efficiency both *in vitro* and *in vivo*, particularly when the ratio of CuBG to PS is lower than 3 (9CB/3PS). This work provided a versatile and scalable approach to enhanced the interaction within inorganic particles and polymer network in hydrogels without extra modification on components.

## Graphical abstract

**Figure:**
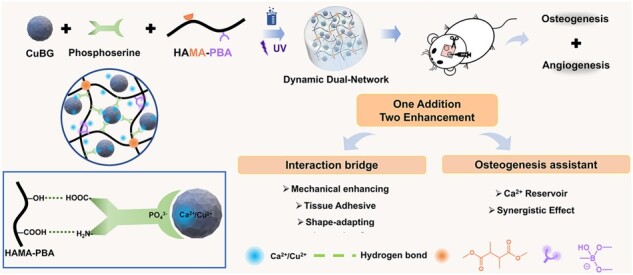

## Introduction

Craniofacial bone defects caused by trauma, infection, tumor and congenital malformation usually requires surgical intervention. There are a large number of demands for clinical treatment, but due to the irregular shape of the defect and thin thickness of skull, the treatment of craniofacial bone defects yet remains an enormous challenge. In order to improve the therapeutic effect, numerous bioactive bone grafts based on osteoconductive inorganic materials like calcium phosphates and bioactive glasses (BG) have been developed [1]. However, commonly used granule-like or bulk scaffolds exhibited operational inconvenience. More recently, bone cements, bone putties [2–4], 3D printing scaffolds [5–9] and hydrogels [10–13], which focused on providing shape matching property, have gained wide interests. Although bioactive bone cements and putties products like NovaBone Putty^®^ have been widely used in clinic, inorganic particles in this system hardly bond with each other and with the liquid phase, which will risk collapse and reduction of the reconstruction effect. Moreover, many organic–inorganic composite hydrogels were prepared but based on poor interaction between the polymer network and inorganic particles, thus failing to address these questions. A combination of superior shape matching performance, suitable mechanical property, excellent osteogenic activity and operational convenience necessitates the rational selection and design of the bioactive materials for bone repair.

BG could stimulate osteogenesis through releasing Si, Ca and other specific doped ions which could activate osteogenesis-related signaling pathways such as Wnt/*β*-catenin, MAPK, ERK and p38, etc., by their individual or synergistic effects [14, 15]. Recent studies have revealed that mesoporous BG (MBG) has a larger specific surface area than traditional BG like 45S5, resulting in higher osteogenic activity [8, 16]. Our previous studies and other research [17–20] have also shown the angiogenetic ability of copper-doped BG, which can improve the efficiency of osteogenesis in its early stage. At the same time, owing to its antibacterial ability, Cu can also inhibit potential infection [21, 22]. Since the high temperature sintering process results in the loss of bioactivity, sintering BG into different shapes or into various forms with special porous internal structures to match the shape and structure of irregular bone defects is usually inadvisable. Therefore, in many studies, BG has been combined with polymers to form composite scaffolds that meet the requirements of craniofacial bone defect repair.

More recently, hydrogels with specific modification can be endowed with injection, adhesion, self-healing and other properties [23–26], which can automatically fill defects of various shapes and form a stable binding with the surrounding tissue. However, in many composite hydrogels, BG or other inorganic particles were added to the network by simple mixing [27–29], which may lead to mechanical defects and limit the content of particles. Hence, the critical issue is how to improve the interaction between polymer network and particles, to ensure that the higher content of particles can distribute in the polymer network more uniformly, which could improve the flexibility and mechanical properties of the hydrogels to meet the requirements for bone defect repair as well as ease of handling. One of the main enhancing strategies is modification on the particles. With amino groups [30], polydopamine [31] and other functional groups modified on the BG surface, uniform distribution of particles and better mechanical properties of hydrogels are achieved via the formation of non-covalent/covalent bonds (e.g. electrostatic interaction or Schiff base reaction [32]) between inorganic particles and polymers. Besides, decorating polymers with specific functional groups that could toughly interact with Ca^2+^ in BG, such as bisphosphonates [33, 34] and carboxyl groups [35] turns to be another option. However, the complicated synthesis process and limited grafting rate of these strategies hinders the clinical application. To solve this problem, we explored a dynamic chemical bridging strategy to construct highly flexible and osteogenic hydrogels for irregular craniofacial bone defect repair, in which phosphorylated amino acid was added as both a crosslinker and an osteogenic assistant by simply tuning the feed ratio.

Combining the methods of dynamic and non-dynamic crosslinking stands out as a favorable access to tune the flexibility and collapse resistance of hydrogel. Given that dynamic phenylborate ester bonds tend to form under an alkaline condition, hyaluronic acid modified with phenylboric acid and methacrylic acid (HAMA-PBA) is proofed to self crosslink into a network with good shape adaptability [36, 37]. Subsequently, covalent crosslinking among methacrylate groups triggered by UV light can provide the anti-collapse property. The phenylborate ester bonds between HAMA-PBA and polysaccharides on cells or tissue also enable the hydrogel to integrate with the fragmented tissue around the defect site [38]. Therefore, HAMA-PBA was chosen to be the polymer network of composite hydrogels.

Substances containing phosphate groups usually have good osteogenic properties, such as bisphosphonates [33], which are usually used to treat osteoporosis. Phosphoserine (PS), as a main constituent of bone sialoprotein (BSP) and osteopontin (OPN), could trigger new bone formation by improving the activity of osteoblast as well as activating BMP and Runx2 signaling pathways [39, 40]. In this way, PS shows potential application in the repair of bone defects by being modified at the backbones of polymers such as collagen or peptides [41–43]. The phosphate groups on PS could combine with Ca^2+^ from CuBG and surrounding environment *in vivo*, providing osteogenic properties. On the other hand, the amino and carboxylate groups on PS could form hydrogen bonds with hydroxyl groups on HAMA-PBA, resulting in a stronger interaction between CuBG and the polymer network. With these properties, PS can be introduced into a composite hydrogel as a dynamic crosslinker, in other words, an interaction bridge, as well as a critical osteogenic regulator to achieve a synergistic effect with CuBG. The properties of hydrogels could be regulated by the ratio of PS and CuBG.

In order to verify whether the CuBG/PS hydrogels meet the clinical demands for craniofacial bone defects repair, we test the tunable mechanical properties, flexibility, antibacterial property and *in vitro* osteogenic activity of hydrogels. A rat critical skull defect model was used to analyze the reconstruction effect of the composite hydrogels *in vivo*.

## Materials and methods

### Materials

Sodium hyaluronic acid (HA) (10 MDa) was provided by Shanghai Yuanye Bio-Technology Co., Ltd (Shanghai, China). L-O-Phosphoserine and methacrylic anhydride (MA) were purchased from Sigma-Aldrich. Luria–Bertani agar (LB agar) and LB broth were purchased from Guangzhou HuanKai Biology Technology Co., Ltd. 2-Morpholinoethanesulfonic acid (MES), 3-aminophenylboric acid (PBA), 4-(4,6-dimethoxy-1,3,5-triazin-2-yl)-4-methylmorpholinium chloride (DMTMM) and all other reagents (unless indicated) were purchased from Shanghai Aladdin Industrial Co., Ltd (Shanghai, China). All assay kits used for the cell experiments were purchased from Beyotime Biotechnology.

### Preparation of copper-doped 58S bioactive glass

The 58S bioactive glass was prepared by a sol-gel method. Its molar composition is 60%SiO_2_–36%CaO–4%P_2_O_5_ and here we named it as 0CuBG. The BG with copper molar ratios of 0.2%, 0.5% and 1% was synthesized with partial replacement of calcium by copper and named as 0.2CuBG, 0.5CuBG and 1CuBG, respectively.

Briefly, after mixing 46.9 ml deionized water and 7.82 ml 1 M HCl solution evenly, 66.1 ml tetraethyl orthosilicate (TEOS) and 6.5 ml triethyl phosphate (TEP) were added slowly and sequentially with continuously stirring. Ca(NO_3_)_2_·4H_2_O and Cu(NO_3_)_2_·xH_2_O were added to obtain CuBG with different degrees of substitution. After aging at room temperature for 2 days, the gel was dried at 60°C and 120°C respectively, for 3 days, to remove moisture. Finally, by a heat treatment at 650°C for 3 h, the final product was obtained.

### Analysis of chemical composition, structure, morphology, and mineralization property of CuBG

FITR (Nicolet, USA) was used to analyze the chemical composition of CuBG and its mineralized products. The test wavelength ranged from 2000 to 400 cm^−1^. The crystal structure of CuBG and its mineral deposition were analyzed by XRD (PANaliytical B.V., Netherlands) in a 2θ range from 10° to 70°, using a Cu-Ka1 radiation tube (voltage of 40 KV, tube current of 100 mA). The pore structure and specific surface area of CuBG were tested by BET (Quantachrome, USA). The degassing of samples was performed for 4 h in a vacuum drying oven at 250°C. The U-shaped sample tube was used to load the powder for test. The adsorbed gas flowed through the sample in liquid nitrogen environment. SEM (JEOL, JSM-5600, Japan) is used to analyze the surface morphology of CuBG.

### The cellular compatibility test of CuBG

In order to test the tolerance of BMSCs to Cu^2+^ concentration and find a suitable Cu doping content in BG, the cell compatibility of the synthesized CuBGs for BMSCs was tested. CuBGs were immersed in the culture medium for 24 h to obtain the extracts at a concentration of 1 mg/ml. About 1 × 10^4^ cells were seeded in 48-well plates and incubated with these extracts for 1, 3, and 5 days, respectively. A CCK-8 kit and a live/dead assay kit were used to evaluate cell viability.

### Synthesis and characterization of HAMA-PBA

First, 1 g of hyaluronic acid was dissolved in deionized water, and 3 ml of methacrylate was added. The pH of the solution was kept between 8 and 8.5 for 3 h by adding NaOH. After 1 day of reaction, the solution was dialyzed and lyophilized to obtain HAMA. Dissolve 1 g HAMA in the 0.1 M MES buffered solution, then after adding 1.4 g 4-(4, 6-dimethoxy-1,3, 5-triazin-2-yl)-4-methylmorpholinium chloride (DMTMM) for activating the carboxyl group and stirring for 30 min, 0.14 g 3-aminobenphenylboric acid was added into the solution. After 24 h of reaction at room temperature, the final product HAMA-PBA could be obtained by dialysis and lyophilization. The chemical structure of HAMA-PBA was detected by ^1^H NMR. Mestre nova was used to analyze and integrate the degree of substitution of PBA and double bonds.

### Preparation of photo-crosslinked dual-network composite hydrogel

LAP photo initiator (0.1 wt%) was added to a PBS solution of 2 wt% HAMA-PBA. Then CuBG and PS were added in different proportions according to Table 1 to ensure that the total content was fixed at 12 wt%, and after proper stirring the composite hydrogels were formed. The photo crosslinked dual-network hydrogels can be obtained after 30 s of ultraviolet irradiation with a wavelength of 365 nm.

**Table 1.** Constituents of every group

Name	CuBG content (wt%)	PS content (wt%)
8CB/4PS	8	4
9CB/3PS	9	3
10CB/12PS	10	2
12CB/0PS	12	0

### Structure analysis, swelling, mineralization, ion release test of hydrogels

The internal morphology and pore structure of the freeze-dried hydrogels were characterized by SEM. For swelling ratio test, the hydrogels were formed into a cylinder with a diameter of 5 mm and a height of 5 mm and immersed in simulated body fluids. At regular intervals, the surface was dried and the weight recorded.

The final swelling ratios of hydrogels were calculated by the following formula: where *W*_t_ represent the weight of hydrogels at time *t* (*t* = 1 h, 2 h, 4 h, 8 h, 12 h, 16 h, 24 h, 48 h.) while *W*_0_ represented the original weight of hydrogels.


$$\text{Swelling ratio}=(W_{t}-W_{0})/W_{0}$$

After immersed in SBF for 1, 2, 7 and 14 days, the hydrogels were collected and lyophilized, the formation of hydroxyapatite was characterized by SEM. At the same time, the supernatant of SBF was retained and an inductively coupled plasma optical emission spectrometer (ICP-OES, PerkinElmer PinAAcle 900T, Germany) was used to determine the content of Ca, Si, P, and Cu released from the colloids.

### Rheological, mechanical, and adhesive properties test of hydrogels

The injectable and adaptive properties of the hydrogels were characterized by extruding the hydrogels with a 23G syringe and injecting it into different shapes of molds. The two hydrogels were cut simultaneously, and then joined together. The joint was pulled after a period to verify its self-healing properties. A rheometer was used to quantitatively characterize the rheological behavior of hydrogels. First, the linear viscoelastic range was characterized by a strain amplitude sweep test, and the self-healing ability of hydrogels was quantitatively characterized by a continuous step-strain sweep under 1% and 350%.

The compressive strength of colloids was characterized at room temperature using a dynamic thermomechanical meter (DMA, Q800DE, TA) at a compressive rate of 3 mm/min. All specimens were prepared in the form of cylinders with a diameter of 5 mm and a height of 5 mm.

The quantitative tissue-adhesive property of hydrogels was characterized by a lap shear test on porcine skin according to our previous work [36]. The qualitative tissue-adhesive property of hydrogels was demonstrated by putting it between two cubic porcine bones or two irregular rat bones for 10 min and then lifting different weights.

### Anti-bacteria ability test of hydrogels

The antibacterial properties of different hydrogels were studied using Gram-positive *Staphylococcus aureus* and Gram-negative *Escherichia coli* as model bacteria. Ten microliters of LB broth bacterial suspension were added into sterile PBS to form a solution at 106 CFU/ml. After co-incubated with hydrogel for 2 h, the solution was added into 990 μl PBS solution for 2-min shaking, so that the viable bacteria were fully re-suspended. Ten microliters of suspension were evenly coated on LB agar and incubated at 37°C for 12 h. Finally, the number of bacteria on LB agar was counted. Bacterial survival rate is calculated as follows:


$$\mathrm{Survival}=\frac{\mathrm{the}\;\mathrm{number}\;\mathrm{of}\;\mathrm{bacteria}\;\mathrm{in}\;\mathrm{experimental}\;\mathrm{group}}{\mathrm{The}\;\mathrm{number}\;\mathrm{of}\;\mathrm{bacteria}\;\mathrm{in}\;\mathrm{control}\;\mathrm{group}}\times 100\%$$

The control group was HAMA-PBA hydrogel group, and the experimental groups were 8CB/4PS, 9CB/3PS, 10CB/12PS and 12CB/0PS hydrogel, respectively.

### Cytotoxicity of hydrogels on BMSCs and hUVECs

Different hydrogels were immersed in the culture medium for 24 h to obtain the extracts. About 1 × 10^4^ cells were seeded in 48-well plates and incubated with these extracts for 1, 3, and 5 days, respectively. A CCK-8 kit and a live/dead assay kit were used to evaluate cell viability and biocompatibility of hydrogels. For CCK-8 assay, the cells were incubated for 1 h, then the optical density values at 450 nm were detected through a microplate reader (Thermo-Fisher, Varioskan Flash 3001). For live/dead staining assay, the morphology and viability of cells were observed by an inverted fluorescence microscope after stained by calcein-AM and propidium iodide (PI).

### Evaluation of osteogenesis and angiogenesis ability of hydrogel *in vitro*

The expression of several specific genes related to osteogenesis and angiogenesis was quantified by reverse transcription-quantitative polymerase chain reaction (rt-qPCR). BMSCs were seeded in 12-well plates at a density of 5 × 10^4^ cells/well and the culture medium was changed with extracts of different hydrogels on the next day. After culturing for 7 or 14 days, the total mRNA was gathered by High Pure Total RNA Micro Kit (Magen) which would be transcribed reversely into complementary DNA (cDNA) using the PrimeScript RT reagent Kit (Takara, Tokyo, Japan). The expression of BSP, COL I, OCN, OPN and Runx2 genes was performed by rt-qPCR with a Maxima SYBR Green/ROX qPCR kit (Applied Biosystems) and a QuantStudio 6 Flex System (Life Technologies). Through normalizing to GADPH by the 2^−ΔΔCΤ^ method, the relative expression of all target genes was quantified.

The qualitative expression of alkaline phosphatase (ALP) was evaluated by an BCIP/NBT ALP Color Development Kit. Briefly, BMSCs were seeded in 24-well plates at a density of 1 × 10^4^ cells/well. After culturing for 7 or 14 days with extracts of different hydrogels, the cells were fixed by 4% paraformaldehyde and stained by the BCIP/NBT for 30 min. The stained cells were observed by a bright field microscope.

The quantitative activity of ALP was evaluated by an ALP Assay Kit and a BCA Protein Assay Kit (Beyotime) after the same cell culturing procedure as above. The activity of ALP in different groups was calculated after the operations guided by the instruction manual of the Assay Kit mentioned.

We used 0CuBG and 0.2CuBG, respectively, with phosphoserine to form hydrogels at the ratio of 8CB/4PS to evaluate the promotion of Cu^2+^ on angiogenesis. The cells of blank control group were cultured by endothelial cell medium (ECM) only. The expressions of eNOS, HIF-α, KDR and VEGF genes which represent angiogenesis were evaluated by 5-day-cultured hUVECs the same way as the osteogenesis related genes we mentioned above.

The recruitment effect of hydrogels on the hUVECs was estimated by 24-well transwell chambers (Corning, USA), hUVECs were seeded in chambers at a density of 8 × 10^4^ cells/chamber, extracts of different hydrogels were added into the well below. After cultured for 12 or 24 h, the cells were fixed by 4% paraformaldehyde and then stained by 0.1% crystal violet. Cells remained on the upper membrane of the chambers were wiped away. Cells migrating through the membrane were observed and calculated by the inverted fluorescence microscope and ImageJ, respectively.

### Evaluation of osteogenesis and angiogenesis ability of hydrogel

Thirty Sprague–Dawley (SD) rats (male, 200–250 g) were purchased from the BesTest Biotechnology Co., Ltd. (Zhuhai, China). All animal procedures were performed following protocols approved by the Ethics Committee, HuaTeng Biotechnology Co., Ltd. The reference number of the ethical approval provided by the Ethics Committee was HTSW220706. Briefly, a critical-size bone defect was established on both sides of each rat’s skull by a driller (5 mm diameter). Comparing to the blank control group, the defect area was injected and filled with 8CB/4PS, 12CB/0PS and 12BG/0PS (the hydrogel containing of 0CuBG), respectively. About 4, 8 and 12 weeks after the surgery, the rats were euthanized with overdosed pentobarbital sodium and the skulls were obtained and fixed with 4% paraformaldehyde. A micro-CT (XTV 160H, Nikon, Japan) was used to quantify the new bone formation. Moreover, the histological changes of osteogenesis and angiogenesis at the defect area were evaluated by hematoxylin–eosin (H&E) staining and Masson trichrome staining. Furthermore, vascular formation was detected by immunohistochemical staining of CD31 and VEGF.

### Statistical analysis

All quantitative results were presented as mean±SD. Comparison of mean values across all groups was analyzed by one-way ANOVA using GraphPad Prism 8. It is considered to be statistically significant when the value of *P* < 0.05. Besides, the ‘*’, ‘**’ and ‘***’ represented *P* < 0.05, *P* < 0.01 and *P* < 0.001, respectively. All experiments were performed at least 3 times to ensure the repeatability of the results.

## Results and discussion

### Characterization of CuBGs and HAMA-PBA

**Figure 1.**
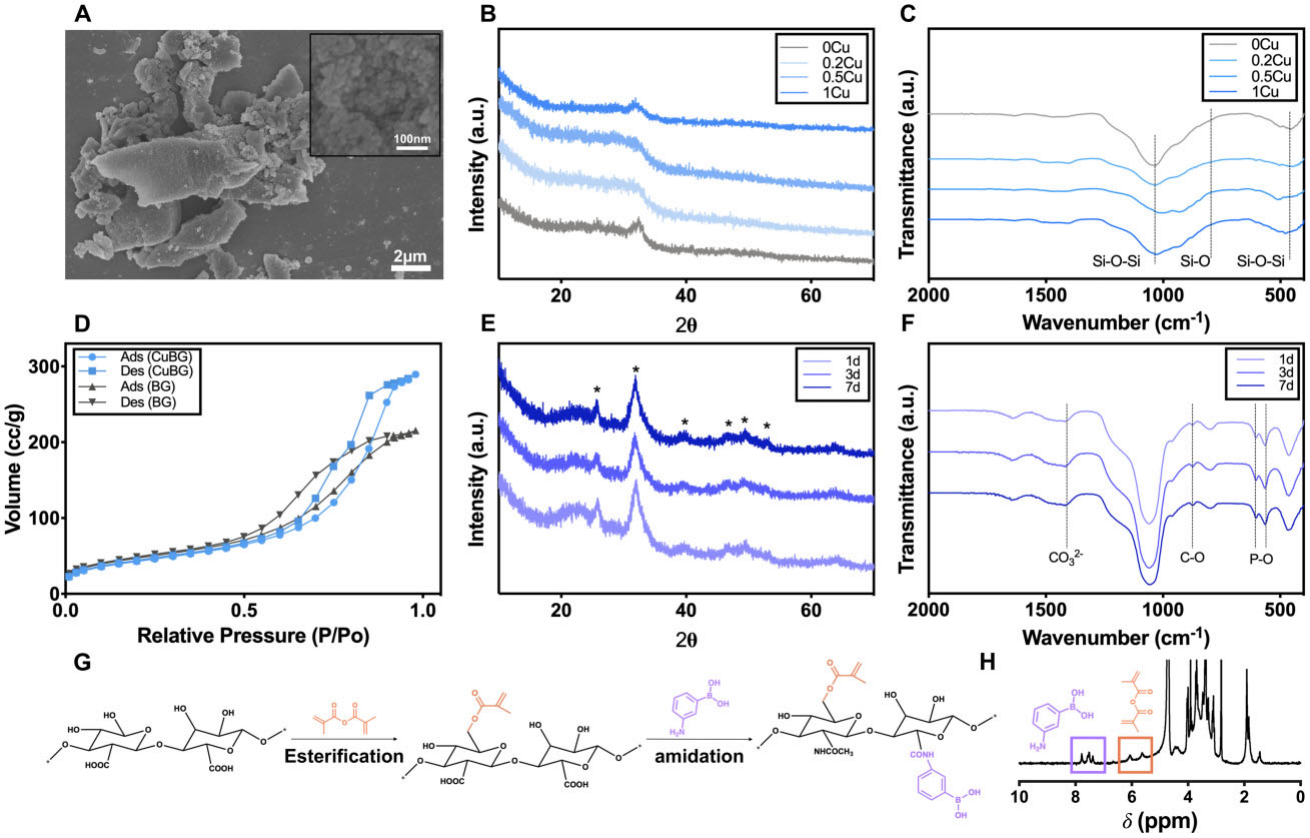
SEM images (**A**) of 0.2CuBG, the small image on the top right (scale bar = 100 nm) is enlarged view of the large image (scale bar = 1 µm). XRD patterns (**B**) and FTIR spectra (**C**) of 0CuBG, 0.2CuBG, 0.5CuBG, 1CuBG. (**D**) Nitrogen adsorption and desorption curves of 0CuBG and 0.2CuBG. (**E**) XRD patterns and (**F**) FTIR spectra of 0.2CuBG soaked in SBF for 1, 3, 7 days. (**G**) Scheme of synthetic route and (**H**) ^1^H NMR spectrum of HAMA-PBA.

Through a sol-gel method introduced in our previous work [44], 58S bioactive glass doped with different content of Cu (0 mol% for 0CuBG, 0.2 mol% for 0.2CuBG, 0.5 mol% for 0.5CuBG, 1 mol% for 1CuBG) was prepared successfully. The contents of Si, Ca, P and Cu in different groups of CuBG were detected by EDS and shown in Table 2, which confirmed the successful doping of Cu into the network of glasses. The SEM images of CuBGs showed its irregular shape (diameter was between 10∼40 µm) and porous surface (Fig. 1A), the enlarged image was presented in Supplementary Fig. S1. Furthermore, a BET test was used to characterize the specific surface area of CuBGs. The nitrogen adsorption and desorption curves (Fig. 1D) were type ‘IV’ isotherm, which confirmed the mesoporous structure observed by SEM images. The specific surface areas of 0CuBG and 0.2CuBG were 169.68 and 155.81 m^2^/g, respectively, showing that the slightly introduction of Cu did not change the mesoporous structure of BG. The XRD patterns (Fig. 1B) showed that all groups of CuBG had a broad diffuse peak between 20° and 30°, suggesting the amorphous structure. A peak at 32° represented a slight crystallization in the heat treatment procedure. The FTIR spectra of CuBGs (Fig. 1C) showed characteristic bands of Si–O–Si group at 475 cm^−1^ (Si–O–Si stretching), 795 cm^−1^ (Si-O stretching), and 1054 cm^−1^ (Si–O–Si bending), which confirmed the formation of Si–O–Si network of BG.

**Table 2.** Different chemical elemental contents of 0CuBG, 0.2CuBG, 0.5CuBG, 1CuBG

Group	Si (%)	Ca (%)	P (%)	Cu (%)
0CuBG	51.69	41.74	6.57	0
0.2CuBG	54.23	37.88	7.69	0.19
0.5CuBG	51.10	39.28	9.34	0.39
1CuBG	50.72	41.45	6.76	1.06

By testing the cytotoxicity of different CuBGs on BMSCs through a CCK-8 kit (see Supplementary Fig. S1), we found that 0.2CuBG showed the lowest cytotoxicity. Therefore, 0.2CuBG was used to form the final composite hydrogels. The mineralization property *in vitro* of 0.2CuBG was also evaluated. After immersing in SBF for 1, 3, 7 days, respectively, the 0.2CuBG was dried and detected by XRD and FTIR to determine the mineral deposition. The characteristic peaks of hydroxyl apatite (HA) at 26°, 32°, 46°, etc., were detected on XRD patterns at every time point (Fig. 1E). Their intensity became higher over time, indicating the lattice structure became more complete. The characteristic bands of PO_3_^2−^ at 577 cm^−1^, 613 cm^−1^ (P–O bending), and 976 cm^−1^ (P–O stretching) as well as the characteristic bands of CO_3_^2−^ at 893 cm^−1^ and 1440 cm^−1^ (C–O bending) on the FTIR spectra (Fig. 1F) represented the deposition of HCA (hydroxyl apatite containing carbonate).

Following the method in our previous work [45], HAMA-PBA was obtained by an esterification reaction and an amidation reaction (Fig. 1G). The ^1^H NMR spectrum of HAMA-PBA (Fig. 1H) confirmed the successful grafting of methacrylate groups and phenylboronic groups on the HA. The characteristic peaks at the range of 5–6 and 7–8 ppm were attributed to protons in vinyl groups and phenylboronic groups, respectively. The degree of substitution of methacrylic acid and phenylboronic acid were 16% and 20%, respectively, which were calculated by comparing the integrated peaks area of them and methyl groups.

### Structure analysis of hydrogel

**Figure 2.**
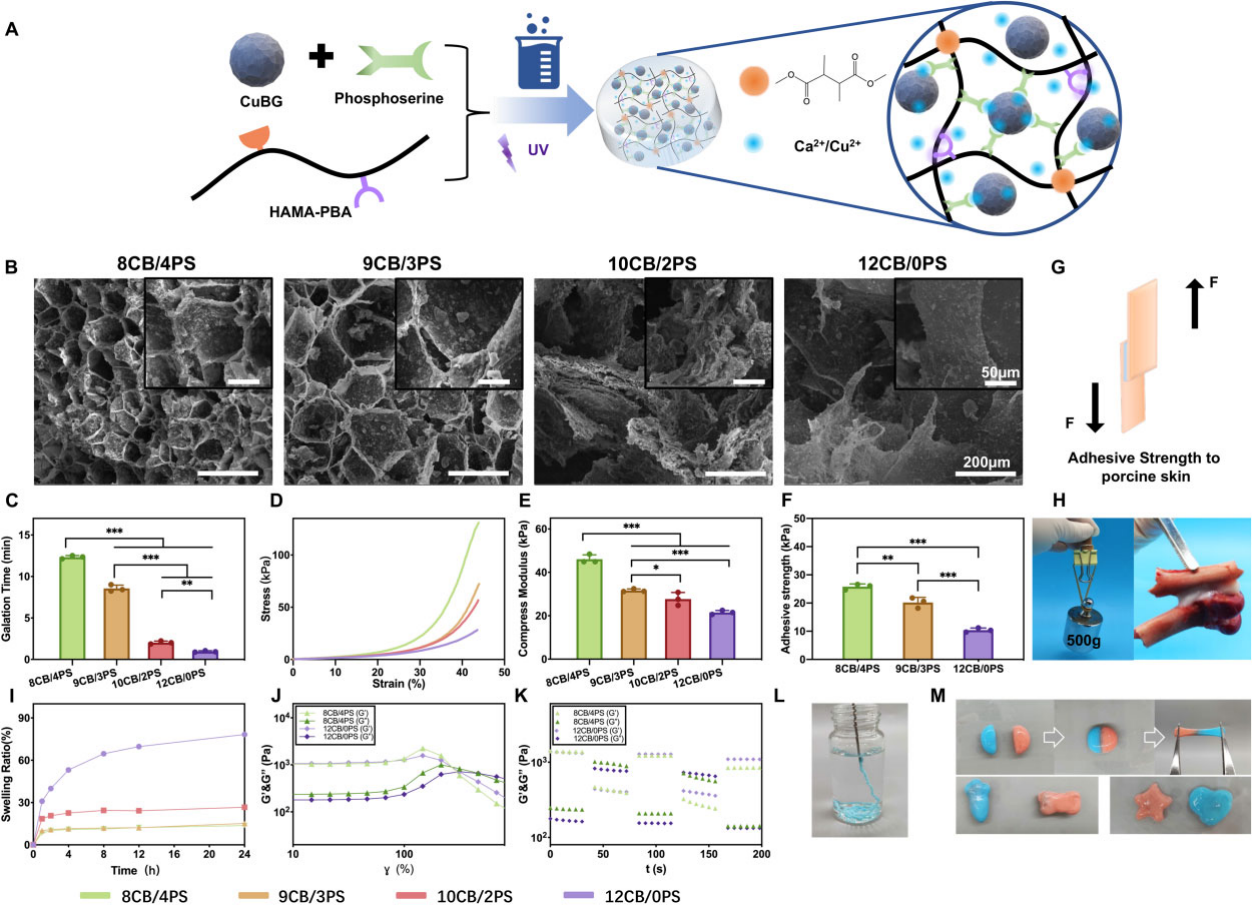
(**A**) The synthetic route and network scheme of hydrogels. (**B**) SEM images of 8CB/4PS, 9CB/3PS, 10CB/2PS and 12CB/0PS hydrogels, the small images on the top right (scale bar = 50 µm) are the enlarged view of the large images (scale bar = 200 µm). (**C**) Gelation time of 8CB/4PS, 9CB/3PS, 10CB/2PS and 12CB/0PS hydrogels. (**D**) Stress–strain curves, (**E**) compress modulus, (**F**) adhesive strength and (**I**) swelling ratio of different hydrogels. (**J**) The strain amplitude sweep test of 8CB/4PS and 12CB/0PS hydrogels at room temperature. (**K**) Self-healing ability of 8CB/4PS and 12CB/0PS hydrogels undergoing continuous step-strain sweep at 1% and 350% at room temperature. The schematic diagram (**G**) of the adhesive strength test of hydrogels to porcine skin. Macrograph of (**H**) the tissue-integrating, (**L**) injectable, (**M**) self-healing and shape-adaptive property of hydrogels.

By one-pot method, CuBG and PS were mixed in PBS solution of HAMA-PBA and fully stirred to form the final product hydrogel. The phosphate groups of PS could combine with the Ca^2+^ released from CuBG. In addition, the amino groups and carboxylate groups of PS could form hydrogen bonds with hydroxyl groups on HAMA-PBA (Fig. 2A). Therefore, the introduction of PS could build a bridge between HAMA-PBA and CuBG instead of a simple mixing of the latter two. It could enhance the mechanical property of hydrogel through forming a denser network. The self-healing property of composite hydrogel was provided by dynamic boronic ester bonds between PBA groups and vicinal diol groups on HAMA-PBA backbone which were promoted by the alkaline ions released from CuBGs, such as Ca^2+^ and Cu^2+^. The gelation time of 12CB/0PS group was 0.96 ± 0.09 min, 10CB/2PS was 2.04 ± 0.19 min, 9CB/3PS was 8.56 ± 0.40 min and 8CB/4PS was 12.34 ± 0.20 min (Fig. 2C). This process would be slowed by introducing the PS since its acidic groups would lower the pH of the system, which would impact the formation of boronic ester bonds. The extension of gelation time may be an advantage for the distribution of CuBG in the hydrogel network so that the mechanical property of hydrogel may be further strengthened. After the dynamic network was formed, the second network based on the radical polymerization of double bonds is formed through the exposure to a UV light for 30 s, which provided the hydrogel with anti-collapse performance.

The SEM images of different groups of hydrogels (Fig. 2B) showed that the density of hydrogel network became higher as the ratio of PS/CuBG increased. The pore diameter of 12CB/0PS was 582.7 ± 90.1 µm, 10CB/2PS was 412.5 ± 38.7 µm, 9CB/3PS was 205.6 ± 20.2 µm, and 8CB/4PS group was 108.8 ± 19.0 µm, indicating a denser network caused by the introduction of PS. Despite the denser network, BG particles could still disperse uniformly in the network of 8CB/4PS and 9CB/3PS hydrogels.

The compress test of hydrogels confirmed the enhanced mechanical property caused by PS. With the higher ratio of PS to CuBG, the compressive strength and compressive modulus of different hydrogels (Fig. 2D and E) could be increased from 28.3 kPa and 21.54 ± 0.93 kPa (12CB/0PS) to 131 kPa and 46.09 ± 2.00 kPa (8CB/4PS). The lower swelling ratio of hydrogel (Fig. 2I) in the group with higher ratio of PS to CuBG (from 78.25% of 12CB/0PS to 13.74% of 8CB/4PS) was also caused by the increased density of hydrogel network. A lower swelling ratio is beneficial for hydrogel maintaining stability thus to avoid breaking the original structure of the defect or squeezing surrounding cells and tissue *in vivo*.

We also evaluated the injectability and self-healing ability of hydrogel through a rheological test and macroscopic characterization. Two 8CB/4PS hydrogels with different color were cut in semicircle shape (Fig. 2M). After being sticked together for 5 min, the contacting area of two hydrogels showed a mixed color and could sustain a stretch by tweezers without breaking, demonstrating an excellent and fast self-healing ability. Through extrusion, the hydrogels could fit in various molds of different shapes like carrot, bone, star and heart in a short period of time, providing a satisfying shape-adaptability for irregular bone defect repair (Fig. 2M). The hydrogel could also be injected through a 23G syringe (Fig. 2L), showing a satisfying injectability for clinical manipulation. The tissue adhesive property of hydrogels was studied through a porcine adhesion test (Fig. 2F and G). With the increase of PS/CuBG ratio, the adhesive strength of hydrogels got higher (from 10.40 ± 0.76 kPa of 12CB/0PS to 25.80 ± 0.99 kPa of 8CB/4PS). After the 8CB/4PS hydrogel was put between two pieces of cubic pig bone for 10 min, a 500 g weight was able to be lifted without separation of the cubic bones (Fig. 2H). The hydrogel could also stick two rat bone with different shape together, suggesting its satisfying tissue-bonding property which would help the implanted hydrogels to anchor the tissue around the defect area *in vivo*.

Furthermore, the sol-gel transition points of 8CB/4PS and 12CB/0PS hydrogels were determined by the characterizing the linear viscoelastic region through a strain amplitude sweep (Fig. 2J). The intersections of storage modulus (G′) and loss modulus (G″) of both hydrogels were approximately located at 300%. Based on the sol-gel transition point we measured, a continuous and alternate strain sweep at 1% and 350% was conducted to quantitatively demonstrate the self-healing ability of hydrogels (Fig. 2K). Under a high strain at 350%, the storage modulus of 8CB/4PS and 12CB/0PS hydrogels vertiginously dropped to 236 Pa and 173 Pa, respectively, which was caused by the breakage of dynamic bonds. After the restoration to the low strain of 1%, the storage modulus of both hydrogels immediately recovered to their original level (both were at 1400 Pa approximately), corroborating the macroscopic phenomenon we observed. The excellent self-healing performance of hydrogels was attributed to the fracture and rapid reconstruction of the dynamic network made of boronic ester bonds. This could simplify the clinical procedures and provide more leeway for doctors’ maneuver.

### Mineralization *in vitro* and anti-bacterial ability mediated by ion released from hydrogels

**Figure 3.**
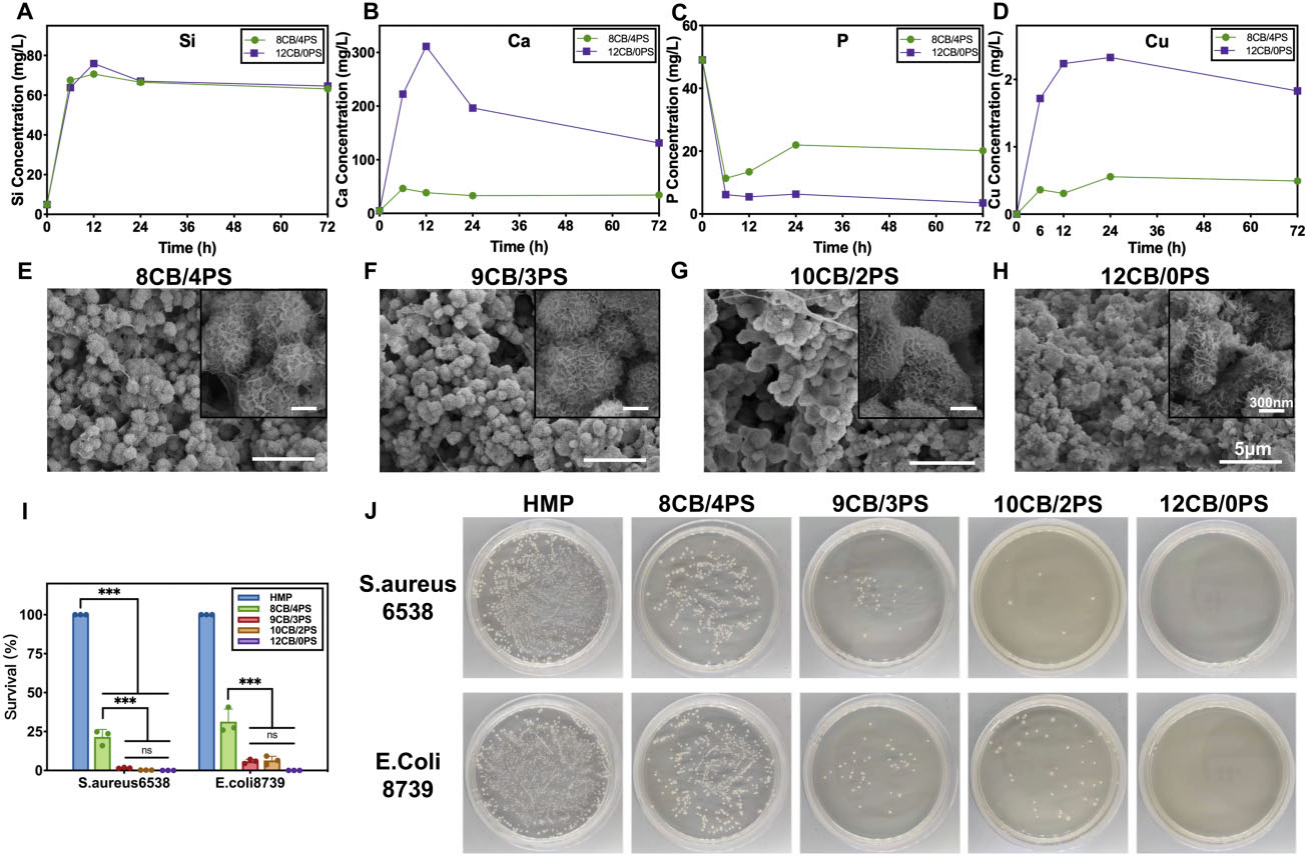
(**A–D**) The concentration of Si, Ca, P and Cu released from 12CB/0PS hydrogel and 8CB/4PS hydrogel detected by ICP. (**E–H**) The SEM images of 8CB/4PS, 9CB/3PS, 10CB/2PS and 12CB/0PS hydrogels. The small images on the top right (scale bar = 300 nm) were the enlarged view of the large images (scale bar = 5 µm). (**I**) The survival rate of two kinds of bacteria (*E. coli* and *S. aureus*) co-cultured with composite hydrogels. (**J**) The macroscopic images of the bacteria colonies on the plates.

The concentration of ions which released from the 8CB/4PS and 12CB/0PS hydrogels after being immersed in SBF for specific time was detected by ICP (Fig. 3A–D). Si, Ca and Cu all rapidly released into SBF in the first 12 h and reached their maximum concentrations. Apart from the effect caused by the content of CuBG in the hydrogels, the content of Ca and Cu released from the 8CB/4PS hydrogel still apparently lower than those from the 12CB/0PS hydrogel, showing the ion interception of PS. After 12 h, the concentration of Ca dropped while the concentration of P dropped after the first 6 h, suggesting that apatite mineralization was taking place.

After the colloids were soaked in SBF for 7 days, the SEM images (Fig. 3E–H) showed the apatite mineralization depositing on hydrogels. All groups of hydrogels had lots of flowers cluster-like mineral deposition overspread their network. Those deposition on 8CB/4PS and 9CB/3PS hydrogels were closer to spheric shape and the size of individual sphere was smaller. The SEM images indicated that the ion interception of PS did not inhibit the mineralization process of hydrogels.

Two model bacteria (*E. coli* and *S. aureus*) were used to evaluate the antibacterial property of hydrogels by a spread-plate experiment. The groups cultured with HMP (HAMA-PBA hydrogel) were set as the control. The survival rate of bacteria was calculated by counting the numbers of bacteria colonies on the plate (Fig. 3I). The mean survival rates of *S. aureus* and *E. coli* co-cultured with 8CB/4PS, 9CB/3PS, 10CB/2PS and 12CB/0PS hydrogels were all less than 20% and 30%, respectively. It can be seen that all composite colloids are able to inhibit two kinds of bacteria from growing (Fig. 3J). In particular, bacteria colonies were barely found on the plate of 10CB/2PS and 12CB/0PS groups, illustrating the promising anti-bacterial property of hydrogels.

### Biocompatibility of hydrogels

**Figure 4.**
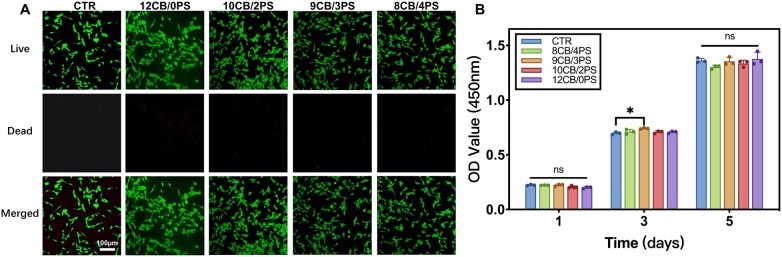
(**A**) The inverted fluorescence microscope images of AM/PI-stained BMSCs after cultured with the extracts of different groups of hydrogels for 24 h, scale bar = 100 µm. (**B**) The optical density at 450 nm of BMSCs after cultured with the extracts of different groups of hydrogels for 1, 3 and 5 days.

Biocompatibility is a critical index to evaluate the biosafety of biomaterials. BMSCs play an important role in bone tissue repair. First, the biocompatibility of hydrogels was evaluated by a live/dead staining assay and demonstrated by inverted fluorescence microscope images, in which the live and dead cells were stained in green and red, respectively. Cells exhibited in a good state with a shape of spindle after cultured with the extracts of different hydrogel for 1 day, and dead cells could be barely found (Fig. 4A). Subsequently, the cytotoxicity of hydrogels was assessed by a CCK-8 assay. BMSCs in all groups proliferated normally when cultured with the extracts for 1, 3, and 5 days, which attested to the fact that all groups of hydrogels had no cytotoxicity and supported cell proliferation (Fig. 4B).

Angiogenesis is essential in the early stage of new bone formation. hUVECs take part in the vascular formation [46]. The angiogenesis stimulation ability of Cu has been proved by many works [17, 19]. Here, we used 0.2CuBG and 0CuBG to form hydrogels at the CB/PS ratio of 8/4, respectively. The results of live/dead staining and CCK-8 assay (see Supplementary Figs S2 and S3) indicated that the presence of Cu did not inhibit the proliferation and viability of hUVECs.

### Osteogenesis of BMSCs and angiogenesis of HUVCs *in vitro* promoted by hydrogels

**Figure 5.**
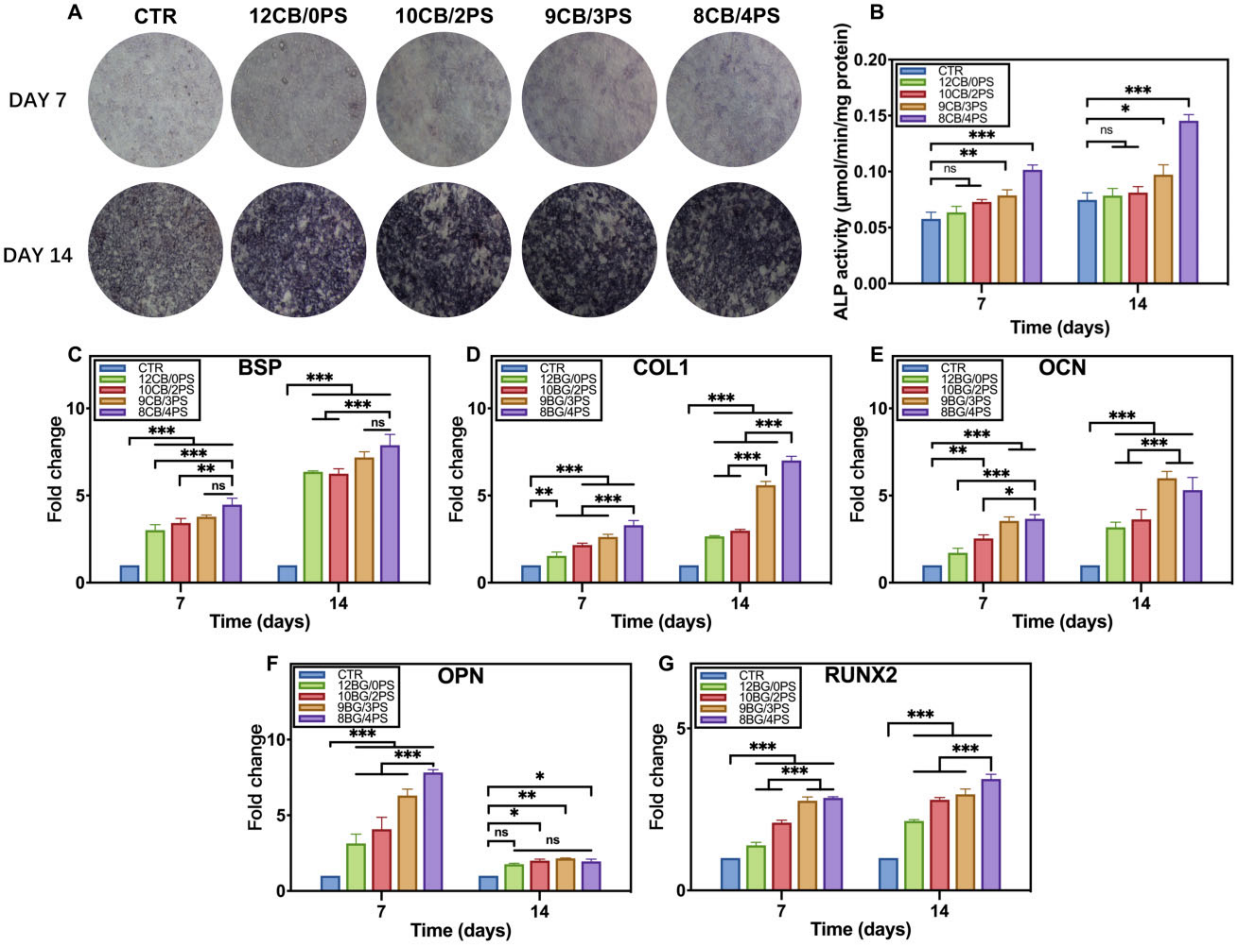
(**A**) The micrographs of BMSCs which were ALP stained after cultured with hydrogels extracts for 7 and 14 days. (**B**) The quantitative result of ALP activity of BMSCs after cultured with hydrogels extracts for 7 and 14 days. The expression of (**C**) BSP, (**D**) COL I, (**E**) OCN, (**F**) OPN, (**G**) Runx2 in BMSCs after cultured with hydrogels extracts for 7 and 14 days.

The osteogenic promotion of colloids at protein level was assessed by ALP staining and its quantitative activity. Staining of ALP became darker but at a different degree as the culture time prolonged (Fig. 5A). Determined from the intensity and darkness of staining, the expression of ALP is significantly enhanced in the groups stimulated by the hydrogels containing more PS, in which the osteogenic stimulation of CuBG is boosted by PS. The quantitative result (Fig. 5B) complied with the qualitative staining, only when PS content was over 3wt%, the enhanced effect would turn significant.

To investigate the synergistic osteogenesis stimulation of CuBG and PS on BMSCs at the molecular level, we performed osteogenic differentiation experiments. The expression of five particular osteogenesis-related genes (BSP, COL I, OCN, OPN, Runx2) was measured by rt-qPCR after the cells were co-cultured with extracts of hydrogels for 7 and 14 days (Fig. 5C–G). Compared with the control group cultured in osteogenic induction medium (complete medium supplemented with 50 ng/ml L-ascorbic acid, 10 mmol/l glycerin β-phosphate 10 nmol/l dexamethasone), osteogenesis-related genes were upregulated in the groups that BMSCs were co-cultured with hydrogel extracts with or without PS, suggesting a significant osteogenic stimulation of the hydrogels. With the higher ratio of PS to CuBG, the expression of these genes got higher. In particular, a more noticeable upregulation of osteogenic genes was found in the group that ratio of CuBG/PS was lower than 3 (9CB/3PS and 8CB/4PS). These results indicated that the osteogenic stimulation was improved by the introduction of PS whereby synergistic regulation and promotion of osteogenesis was achieved in combination with CuBG.

**Figure 6.**
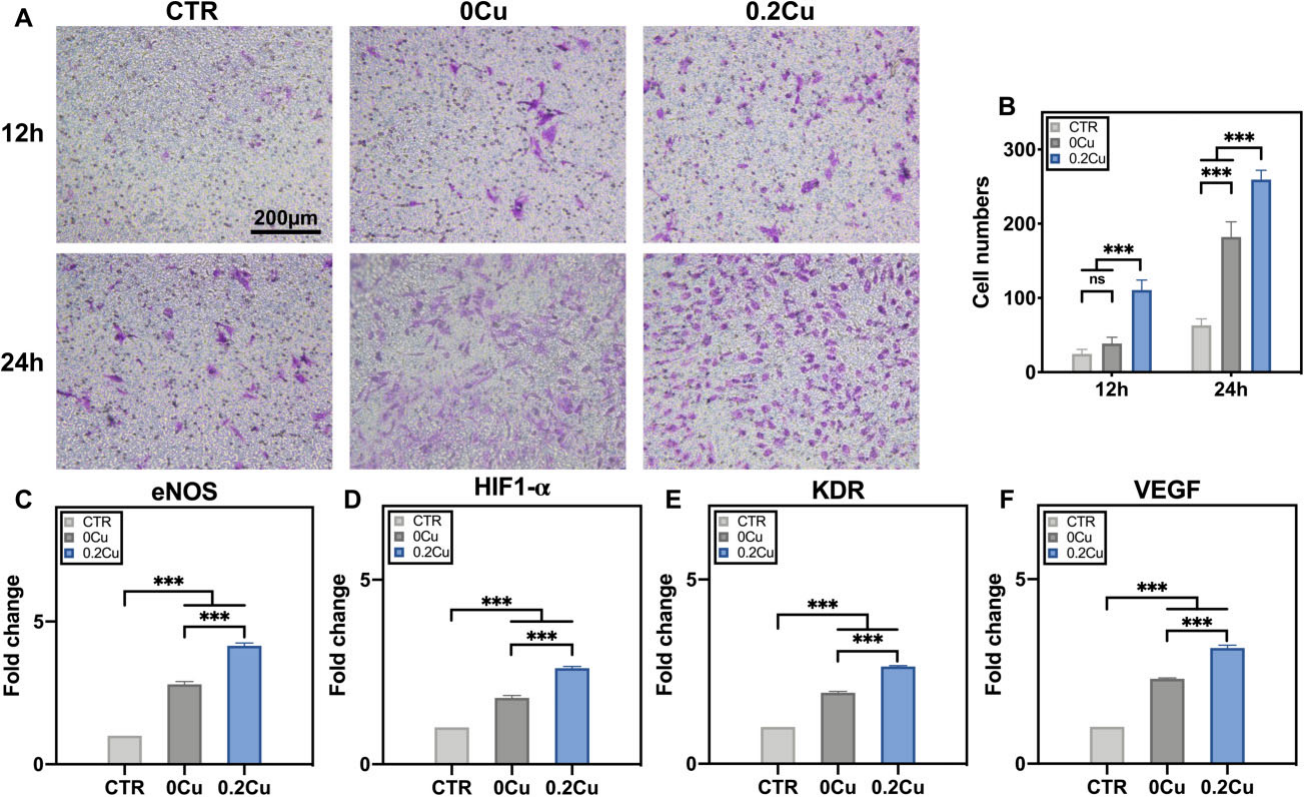
The (**A**) micrographs and (**B**) number of hUVECs migrated through the transwell membranes after co-cultured with the ECM, 0CuBG hydrogel and 0.2CuBG hydrogel, respectively, for 12 and 24 h, scale bar = 200 µm. The expression of (**C**) eNOS, (**D**) HIF1-α, (**E**) KDR and (**F**) VEGF in hUVECs after co-cultured with the ECM, 0CuBG hydrogel and 0.2CuBG hydrogel respectively for 5 days.

The angiogenetic stimulation of CuBG in hydrogels was evaluated at both molecular and cell level. 0CuBG and 0.2CuBG were respectively applied to form the hydrogels used for further assessment at a ratio of 8CB/4PS. Endothelial cells could form microcapillary through migration and proliferation [46], which is controlled by the expression of specific genes (eNOS, CD31, VEGF, etc.) and the stimulation of related signaling pathways (Notch, TGF-β, BMP, etc.) [47–50]. The transwell experiment was taken to estimate whether Cu^2+^ releasing from hydrogels could control the behavior of hUVECs for further angiogenesis. The crystal violet staining (Fig. 6A) indicated that 0CuBG hydrogel could recruit hUVECs. As we expected, in the group of 0.2CuBG hydrogel, more hUVECs migrated to the lower chamber. The quantitative analysis of migrated cells was calculated by ImageJ. hUVECs were more highly recruited at a number of 110 ± 13 and 259 ± 12 after co-cultured with 0.2CuBG hydrogel for 12 h and 24 h, respectively, compared with the 0CuBG group (38 ± 9 and 182 ± 21, respectively) and the control group (24 ± 6 and 63 ± 9, respectively) (Fig. 6B).

The expression of angiogenetic related genes was evaluated through rt-qPCR after hUVECs were co-cultured with extracts of 0CuBG and 0.2CuBG hydrogels for 5 days. Comparing to blank control group (cultured with ECM only), the expression of eNOS, HIF1-α, KDR and VEGF (Fig. 6C–F) was all enhanced by the stimulation of BG whose effect was far more significant with Cu-doped.

### Osteogenesis and angiogenesis stimulated by hydrogels *in vivo* evaluated by a rat critical skull defect model

To evaluate the osteogenic and angiogenetic stimulation of composite hydrogels, a critical skull defect model was made in 30 SD-rats. Based on the considerable antibacterial property and optimum osteogenic stimulation presented in the results above, the 8CB/4PS hydrogel was selected and implanted into a skull defect with a diameter of 5 mm. Besides, to further study the osteogenic and angiogenetic effects of PS and CuBG from the hydrogels, the 12CB/0PS and 12BG/0PS (formed by 0CuBG and PS at the ratio of 12/0) hydrogels were also implanted into a skull defect contrastively. The defect without treatment served as the control group. All groups of hydrogels did not have auxiliary fixations. After 4, 8 and 12 weeks, the rats were euthanatized and further assessments of skulls were taken.

**Figure 7.**
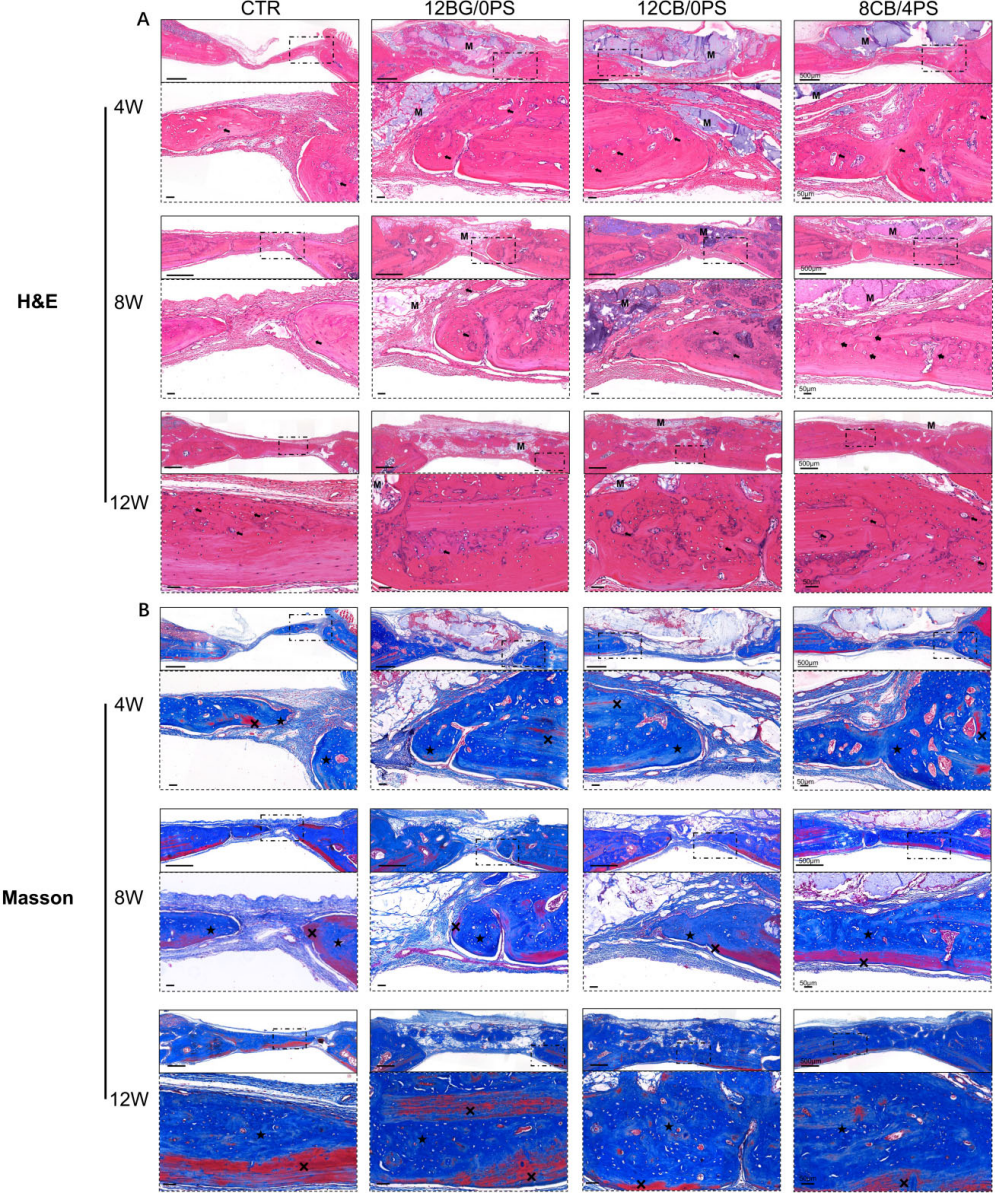
The overall images (scale bar = 500 µm) and enlarged view (scale bar = 50 nm) (whose view field were framed by dotted lines in the overall images) of (**A**) H&E staining and (**B**) Masson’s trichrome staining of new bone formation after 4, 8, 12 weeks post-surgery. The ‘**×**’ indicates the collagen deposition, the ‘★’ indicates the new formed bone, the ‘M’ indicates the implanted materials, the black arrows indicate possible vascular tissue.

H&E staining and Masson’s trichrome staining (Fig. 7A and B) were used to histologically analyze the osteogenic process. All groups of hydrogels did not displace from the defect area while toughly connecting with surrounding bone tissue, indicating the bone tissue integration property of hydrogels. Both H&E staining and Masson’s trichrome staining images clearly showed that the residual hydrogel became less over time, while the spaces were filled with the new bone, suggesting the process of bone regeneration was simultaneously accompanied with the degradation of hydrogels *in vivo*. The degradation of 8CB/4PS hydrogel was faster than other hydrogels, suggesting the content of PS may regulate the degradation rate to match the new bone formation process better. In the comparison with the control group, thicker bone layer was form in all hydrogel-treated groups. The thickness of the new bone got larger with recovery time. There were lots of osteocytes at the intersection of new bone and materials, while some lined up neatly at the mature area. New bone had grown inside the hydrogels over time, showing a satisfying biocompatibility of hydrogels. Tubular vascular structure could be observed at the defect area, especially in 12CB/0PS and 8CB/4PS group, indicating that Cu participated in angiogenetic promotion. The new bone and collagen deposition in Masson trichrome staining pictures were colored in blue, which turned thicker and darker over time. The red staining represented host bone at the early reconstruction stage and the mature regenerated bone at the late stage. Plenty of well-arranged lamellar bone structure was observed from the staining images in all hydrogel groups after 12 weeks post-surgery.

**Figure 8.**
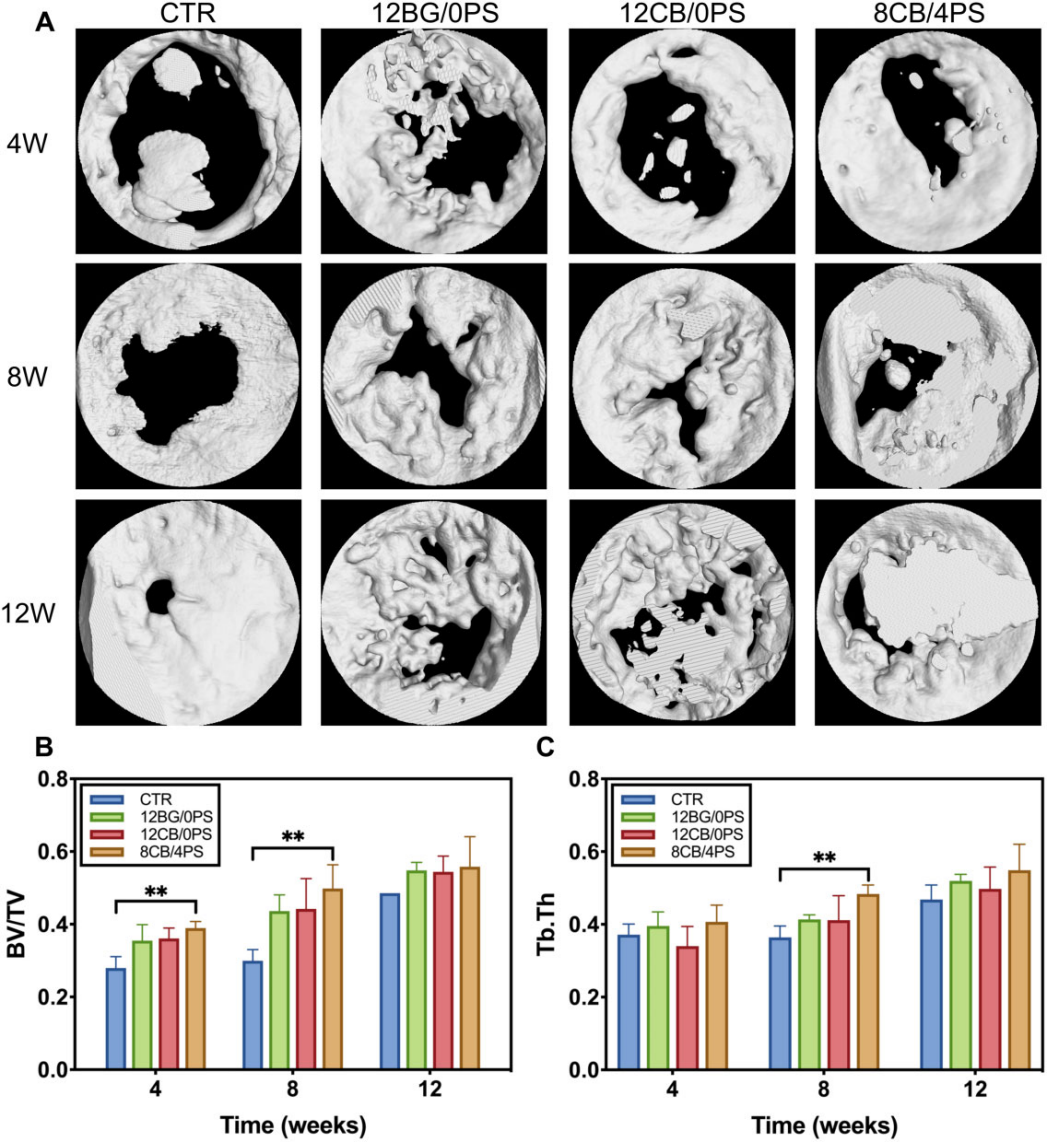
(**A**) Micro-CT reconstruction images of SD rats’ skull defect area. Quantitative analysis of (**B**) new bone volume/total volume and (**C**) trabecular thickness of regenerated bone.

The new bone formation was evaluated by micro-CT. The reconstruction pictures (Fig. 8A) showed that lots of new bone had grown in the defect area of all groups over time. After 12 weeks, the defects were almost completely filled with new bone. New bone in hydrogel groups was thicker than that in control group, indicating a better new bone forming status. The uneven surface was regarded as a result that osteocytes and other osteogenesis-related cells grow into the hydrogels and then formed new bone *in situ*. The quantitative characterization of new bone was further calculated. The new bone volume/total volume (BV/TV) of each group was higher than control group (Fig. 8B), and significant difference was found between the 8CB/4PS group and the control group at 4 and 8 weeks. Trabecular thickness (Tb. Th) (Fig. 8C) was also measured to assess the new bone formation. At 8 weeks, there were significant difference between the 8CB/4PS group and the control group. Two measured indexes suggested that composite hydrogels mainly played a role of osteogenic stimulation in the early-middle process of bone regeneration.

**Figure 9.**
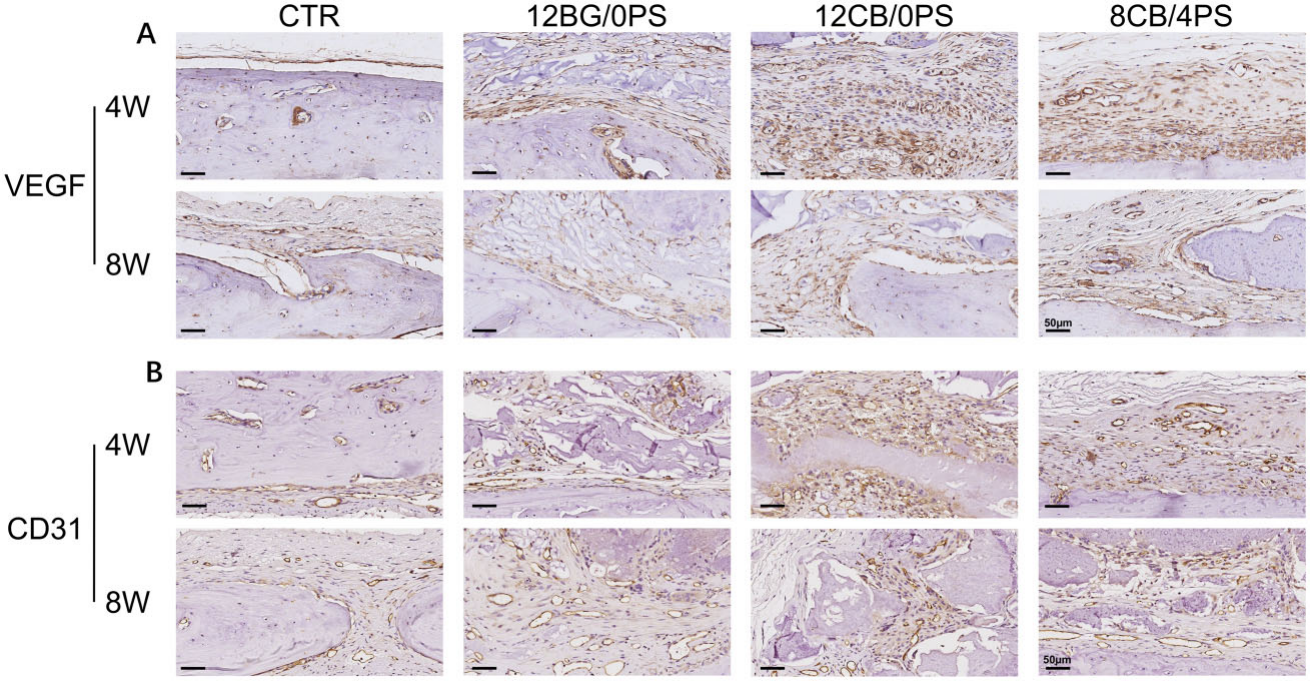
The immunohistochemical staining pictures (scale bar = 50 µm) of (**A**) VEGF and (**B**) CD31 expressed in different groups of hydrogels and the control group after 4- and 8-weeks post-surgery.

Further assessment of angiogenesis *in vivo* was taken through immunohistochemistry (IHC) staining of CD31 and VEGF (Fig. 9A and B). The expressions of CD31 and VEGF in 8CB/4PS and 12CB/0PS hydrogel groups were apparently much more than those in the other two groups, indicating the angiogenetic stimulation of Cu. The expressions of CD31 and VEGF were higher in the area around the implanted hydrogels. Not surprisingly, since the 12CB/0PS hydrogel had more content of CuBG than 8CB/4PS hydrogel, the angiogenesis related IHC staining of which is darker. The VEGF related staining turned light at 8 weeks postoperatively, because the angiogenesis mainly happened in the early stage of bone reconstruction and was supposed to finish in 8 weeks.

## Discussion

In this study, we demonstrated a simplified strategy to enhance the interaction between particles and polymer network in hydrogels, which could be extended to other similar systems. The introduction of PS, playing a bridge role, enhanced and enriched the dynamic combination of CuBG and HAMA-PBA to endow the hydrogel with a higher mechanical property. The gelation process would be slowed by introducing PS since its acidic groups would lower the pH of the system, which would slower the formation of boronic ester bonds. The extension of gelation time may be better for the distribution of CuBG in the hydrogel network, which is another reason for the enhanced mechanical property. The process would also make the hydrogel more flexible so as to quickly adapt to various shape as we showed. Compared with the work that PS was modified onto the polymers but barely had effect on the rheology and mechanical properties of hydrogels [52, 53], our work showed a simpler but effective method.

Through a rapid ion release and exchange, an HCA (hydroxyl apatite containing carbonate) layer could be found on the surface of BG, which was conducive to integrate with the local bone tissue and achieve subsequent bone reconstruction. *In vivo* or in SBF, most of the ions will be released from BG in the first few hours due to high solubility of BG. But it may cause ions loss through the transportation of body fluid in the defect area *in vivo*, which would reduce the bioactivity as well as tissue reconstruction effect of BG [51]. However, the denser network of hydrogel caused by the introduction of PS may make the ions release in a slower manner and finally help to maintain the ion concentration at a higher level. In addition, incorporated PS endowed the hydrogels with lots of phosphate groups which can attract more Ca^2+^ from surroundings. As a result, the composite hydrogel could reserve the ions from CuBG while continuously attracting more Ca^2+^ from body fluids. In this way, the hydrogel served as a mineral ion reservoir for apatite mineralization and new bone formation. On the other hand, the releasing ions from BG will increase local osmotic pressure and pH value in a short period, causing a rupture of the bacterial membrane. In addition, oxidative stress and DNA degradation caused by Cu^2+^ could inhibit bacteria growth more effectively [52, 53], providing a satisfying anti-bacterial ability for composite hydrogels. However, a high pH microenvironment would also cause harm to cells. Hence, although the 12CB/0PS and 10CB/2PS hydrogels showed fascinating antibacterial property (nearly 0% survival bacteria), we chose 8CB/4PS hydrogel with a gentle ions release behavior, which could also inhibit most of the bacteria growth without sharply increasing the pH, for further evaluation *in vivo*. The experiment that 8CB/4PS hydrogel could stick two pieces of cubic bone or two porcine skins tightly together verified the potential tissue bonding property of the hydrogel. It is reasonable to infer that this property attributed to the covalent interaction (e.g. boronate ester bond and amide bond), as well as non-covalent interaction (e.g. hydrogen bond and chelating interaction with Ca^2+^) between bone tissue and hydrogel was mainly donated by the PBA and PS.

There was little research focusing on the synergistic effect of BG and PS or other phosphorylated amino acid, so it is necessary for us to study the mechanism of this synergistic effect. In this work, PS could cooperate with CuBG to stimulate the differentiation of BMSCs for subsequent osteogenesis while the ratio of CuBG to PS needed to be lower than a specific level (3/1) to guarantee a significantly improved property. As we could observe, the 8BG/4PS group showed a best osteogenic ability. OPN could attract macrophages and regulate the activity of osteoclast [54], which mainly works in the early stage of bone formation. Compared to day 7, the difference between the OPN expression of each group at day 14 turned less pronounced, which shows composite hydrogel could stimulate OPN expression and therefore control the behaviors of osteoclast at the early stage of new bone formation. Further evaluation of hydrogels’ osteogenic property *in vivo* showed that PS could enhanced osteogenesis comparing to hydrogel which only contained CuBG. As an essential role in osteogenesis process, angiogenesis would reconstruct blood vessels network which could sustain the supplement of nutrients, oxygen as well as the transport of secretions [46, 55]. The angiogenetic property of Cu is achieved by activating HIF1-α signaling pathway through inducing a hypoxia microenvironment [17, 19]. Our results verified that CuBG in hydrogels exhibited a more powerful angiogenetic stimulation both *in vitro* and *in vivo* compared with BG free of Cu.

## Conclusion

In summary, we designed a composite hydrogel for irregular craniofacial bone defects repair through forming a dual-crosslinked network, in which the dynamic boronic ester bonds provide an injectable, moldable, self-healing property and UV-triggered polymerization among double bonds provides an anti-collapse property. PS and CuBG could not only act as crosslinker to improve the mechanical properties of hydrogel, but also synergistically stimulate osteogenesis *in vitro* and *in vivo*. A rat critical skull defect model verified the co-work of CuBG and PS in composite hydrogel could improve bone regeneration efficiency. This work may provide an application scenario that enables phosphorylated amino acids to perform multiple roles, as well as a simple way to enhance the interaction between particles component and polymer network. The hydrogel could help to simplify clinical manipulation through providing more leeway for the maneuver of doctors while maintaining satisfying osteogenic property.

## Supplementary Material

rbad054_Supplementary_Data
