# Supplementary material for: Phosphoserine enhanced Cu-doped bioactive glass dynamic dual-network hydrogel for craniofacial bone defect repair
Source: Regen Biomater. 2023 May 17;10:rbad054. doi: 10.1093/rb/rbad054 (PMC10243839; doi:10.1093/rb/rbad054)
Supplement: rbad054_Supplementary_Data [file rbad054_supplementary_data.zip › Supplementary Data.docx]

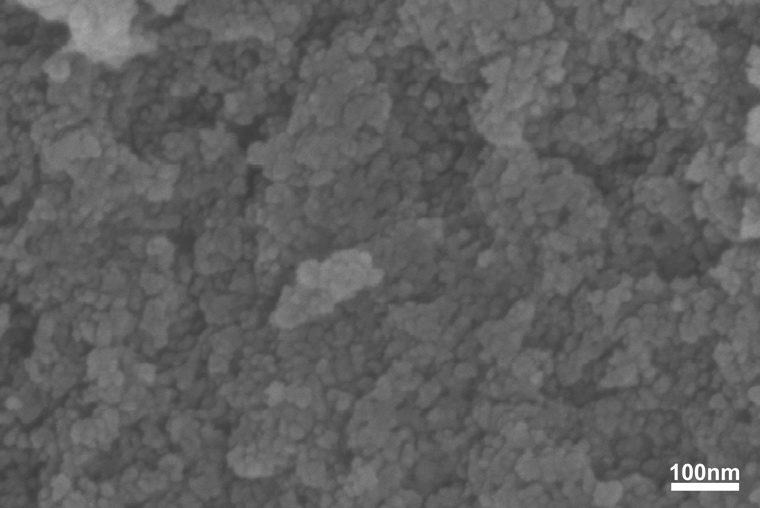


Supplementary Figure 2. SEM images of 0.2CuBG (scale bar = 100 nm).


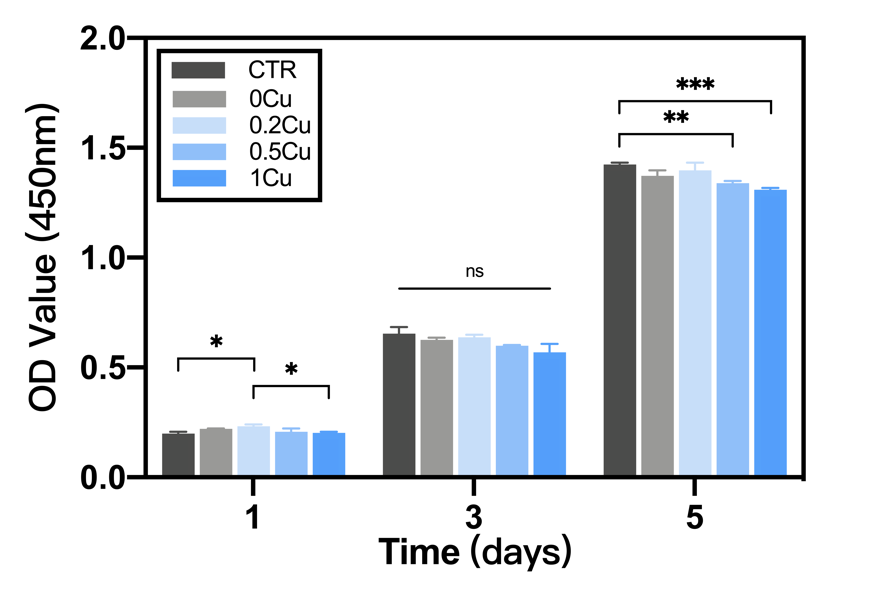


Supplementary Figure 2. The optical density at 450nm of BMSCs after cultured with the extracts of different Cu-doped content Bioactive glass


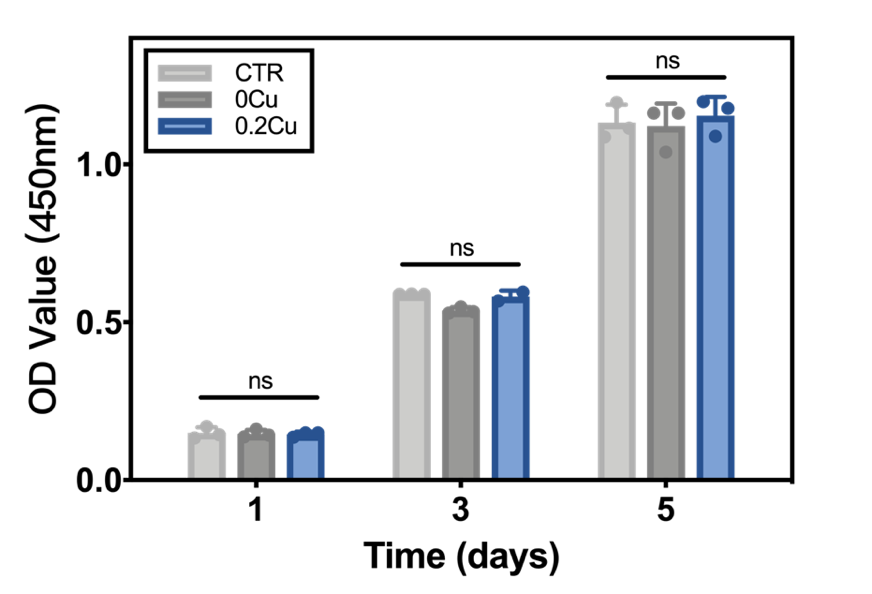


Supplementary Figure 3. The optical density at 450nm of hUVECs after cultured with the extracts of 0CuBG and 0.2CuBG hydrogels comparing to blank control group for 1, 3, and 5 days.


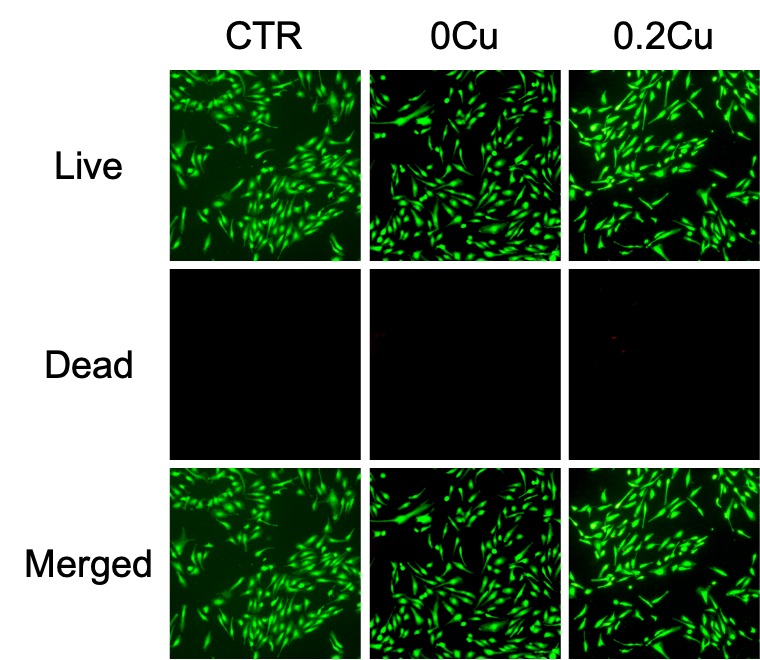


Supplementary Figure 4. The inverted fluorescence microscope images of AM/PI-stained hUVECs after cultured with the extracts of 0CuBG and 0.2CuBG hydrogels comparing to blank control group groups of hydrogels for 24 hours.
