# Supplementary material for: Phosphoserine enhanced Cu-doped bioactive glass dynamic dual-network hydrogel for craniofacial bone defect repair
Source: Regen Biomater. 2023 May 17;10:rbad054. doi: 10.1093/rb/rbad054 (PMC10243839; doi:10.1093/rb/rbad054)
Supplement: rbad054_Supplementary_Data [file rbad054_supplementary_data.zip › Liu response to the reviewers.docx]

Reviewer: 1

Comments to the Author

In this article, the authors fabricated CuBG/PS composite hydrogel, which possessed good properties of injectability, self-healing, adhesive. The authors also showed that the composite hydrogel could be applicable for the calvarial bone defects. This work is significant with potential clinical applications. Before officially accepted, this manuscript has a few points that should be clarified.

1.Why is the effect of PS physical addition better than that grafting PS onto the polymer? Could the authors explain it clearer?

Response: Good question! Although covalently grafting PS in hydrogels can furnished the hydrogel with specific property stably, the maximized osteogenic effect and dynamic integration properties of PS is potentially subject to the complicated synthesis process and limited grafting rate. Besides simplifying the preparation process, physical mixing can provide sufficient PS and free phosphate groups for hydrogel to achieve dynamic integration and enhancement of osteogenic properties by simply tuning the feed ratio. The text has been slightly modified for better clarity (lines 91-97, 606-608).

2.What is the main mechanism of the hydrogel adhesive properties? How do PS and PBA work in the hydrogel?

Response: Thank you for your thoughtful question. The adhesion mechanism of hydrogel consists of two parts. First, the phenylboric acid group in hydrogel can form hydrogen bond with amino groups on the tissue surface, and can also combine with vicinal diol groups on the tissue surface to form phenylborate ester bond. After the introduction of PS, the pH of hydrogel decreased. Thus, there are more dissociative phenylboric acid groups in hydrogel, which can provide more sites to react with tissue surface for adhesive strength enhancement. On the other hand, phosphate group on PS can interact with Ca^2+^ in bone tissue. Meanwhile, amino group and carboxyl group on PS can form hydrogen bond with amino group and carboxyl group on tissue surface. Through the interactions between the hydrogel and the tissue we mentioned above, the hydrogel can realize adhesion with various tissue surfaces. We also added the description in Discussion (lines 630-636).

3. Tissue-bonding and tissue-integrating are different. The authors need to identify an accurate statement.

Response：Thank you for your suggestion! Here we intended to describe the tight adhesion of hydrogels to tissue through the formation of various interactions, so we used the word ‘tissue-integrating’. However, this word may be more suitable to describe the integration of tissue and materials which occurs in new bone formation. The description of tissue adhesion should be tissue bonding. Sorry for the inaccuracy of statement. We have carefully checked the manuscript and revised all statements regarding tissue bonding.

4. In “3.6 in vivo experiment result”, why did the authors choose 8CB/4PS, 12CB/0PS and 12BG/0PS as the experimental groups?

Response：Thank you! In order to exclude the influence of total solid content on hydrogel properties, we fixed the total content at 12 wt%. We set a hydrogel group which was made of 0CuBG, named as 12BG/0PS, as a comparison for investigating the angiogenesis effect of Cu in other experimental groups. To estimate the synergistic effect of PS on osteogenic property of CuBG, we set up a hydrogel group without PS (12CB/0PS), and a hydrogel group containing PS (8CB/4PS), which had been verified to have the best osteogenic effect. The text has been slightly modified for better clarity (lines 531-536).

5. In the Masson’s trichrome staining of Fig. 7, the indication of ‘✕’ and ‘★’appears to be unclear. Please recheck it.

Response：Thank you for your correction. We have carefully checked the figure of Masson’s trichrome staining and revised all indications.

6. Why did the author prefer 58S bioactive glass to nano BG particles? What is the advantage of 58S doped with Cu?

Response：Great question! Although nano-glass particles have higher surface area and multi-functionality, the low Ca content, and the doubts on the clinical use of nano-particles may become the obstacles in products transformation. 58S at micron grade has a mild preparation process with a high Ca content, which also means good osteogenic activity. Given these advantages, 58S at micron grade may be a better choice in clinical product transformation. Vascular network reconstruction is a key process in the early stage of osteogenesis and should be considered in the design of bone repair materials. The osteogenic effect of Cu has been confirmed by a lot of literatures. Our previous work also confirmed that Cu-doped BG has a good osteogenic effect (Biomaterials Science 2021, 9, 5519-5532; ref. 17). Thus, Cu-doped 58S was selected as the coupling material of osteogenesis and osteogenesis.

Reviewer: 2

Comments to the Author

This manuscript entitled "Phosphoserine Enhanced Cu-doped Bioactive Glass Dynamic Dual-network Hydrogel for Craniofacial Bone Defect Repair" demonstrated a series of hyaluronic acid composite hydrogel containing Cu doped bioactive glass (CuBG) and phosphoserine (PS), which have good functions of injectable, tissue-adhesion, anti-bacterial, osteoinduction. A minor revision is necessary before the formal acceptance.

1. Could the hydrogel still self-heal after UV-induced covalent crosslinking? If it can not, what is the advantage of the design of dynamic network?

Response：Good question! The self-healing performance would be weakened after photo-crosslinking since the molecular chain movement in hydrogel would be limited. However, the self-healing property mainly plays a role in filling and adapting irregular defects in practical applications. Once the hydrogel has perfectly adapted to the shape of the defect, the doctor can fix the hydrogel by photo-crosslinking without further adjustment. Therefore, and the self-healing property shows less necessary after crosslinking.

2. Did the authors examine the biodegradability of hydrogel in vivo? What happened to hydrogel after being implanted into the rat skull defect?

Response：Thank you for your question! The degradation progress of hydrogels *in vivo* can be observed by H&E and Masson’s trichrome staining. In staining pictures, hydrogels appeared in white, whose are decreased with time. The 8CB/4PS group showed the fastest degradation rate among all groups. Various enzymes played a role in the gradual degradation of polymer network. At the same time, with the release of Ca^2+^, Si^4+^, Cu^2+^ from CuBG into the surrounding environment, the hydrogel network was further disintegrated. During this process, osteocytes gradually entered the hydrogels and formed new bone, replacing the original defect. Related description has been presented in the revised manuscript (lines 548-554, 556-558).

3. The evaluation of the angiogenetic property of Cu seems to be inadequate. Could the authors explain it more detailed?

Response：Thanks! Our previous work has confirmed the angiogenesis property of CuBG through characterization of angiogenesis-related signaling pathways and proteins expression. It has been found that CuBG enhanced angiogenesis by activating the proinflammatory environment and the HIF-1α pathway of hUVECs. (Biomaterials Science 2021, 9, 5519-5532; ref. 17) Therefore, in this work, we only verified the the angiogenic effect of Cu on related genes expression and cells behavior in vitro (Fig. 6). In the transwell experiment, more hUVECs were recruited in the group treated with the extract of hydrogel containing CuBG. The expression of eNOS, CD31 and VEGF of hUVECs in this group were also remarkably enhanced. What’s more, as shown in Fig. 9, compared with 12BG/0PS hydrogel group (formed by 0CuBG and PS), the expression of angiogenesis-related protein (CD31 and VEGF) is also enhanced in 8CB/4PS and 12CB/0PS hydrogel groups. Based on these results, we believe the data we have presented are able to support the point that Cu has angiogenetic property.

4. The authors entitled the addition of phosphoserine as a ‘dynamic chemical bridging strategy’. Could the authors comment on the meanings and advantages of this strategy?

Response：Thank you for your question! Since phosphate groups on PS could combine with Ca^2+^ from CuBG, physical mixing of PS can provide more phosphate groups to achieve dynamic integration and enhancement of osteogenic properties. On the other hand, the amino and carboxylate groups on PS could form hydrogen bonds with hydroxyl groups on HAMA-PBA, resulting in a stronger interaction between CuBG and the polymer network. With these properties, PS can be introduced into a composite hydrogel as a crosslinker (in other words, an interaction bridge) to enhance the interaction between CuBG and the polymer network. The related description has been presented for clarity (lines 114-121). Such a method can provide injectable, moldable, self-healing and osteogenic property for hydrogels by a simpler synthetic route.

5. In the evaluation of antibacterial property of hydrogels, the antibacterial performance of 8CB/4PS hydrogel was less satisfying than others. Why did the authors still choose it for later experiments in vivo?

Response：Excellent question! The releasing ions form BG will increase local osmotic pressure and pH value in a short period, causing a rupture of the bacterial membrane. In addition, oxidative stress and DNA degradation caused by Cu^2+^ could also inhibit bacteria growth. However, a high pH microenvironment would also cause harm to cells. Hence, although the 10CB/2PS hydrogels showed fascinating antibacterial property (nearly 0% survival bacteria), we chose 8CB/4PS hydrogel with a gentle ion release behavior, which could also inhibit most of the bacteria growth without sharply increasing the pH, for further evaluation in vivo (lines 626-630). Besides, based on the in-vitro experiments, the 8CB/4PS hydrogel had an optimum osteogenic stimulation. To further verify the synergistic osteogenic effect of CuBG and PS, the 8CB/4PS hydrogel was chosen for the contrastive study with 12CB/0PS in vivo. The text has been slightly modified for better clarity (lines 530-536).

Reviewer: 3

Comments to the Author

The work performed in this manuscript is interesting. I appreciate the idea of this research executed. However, some minor comments given below need to be resolved. Manuscript can be more improved in terms of explanations of each outcome.

1. In Abstract author has written that hyaluronic acid was modified by double bonds. Please mention what kind of double bonds were involved in the construct.

Response：Thank you for your reminder! The double bond type is methacrylate-based carbon-carbon double bond. The text has been slightly modified for better clarity (lines 30).

2. Please check the quality of English language.

Response：Thank you for your suggestion! We have carefully checked the manuscript and corrected grammatical errors.

3. Author should give details of parameters used for analysis such as FTIR, XRD, BET and SEM.

Response：Good advice! We have added the parameters for analysis to the revised manuscript (section Materials and Methods). The details of parameters for different tests show as follow:

BET: The degassing of samples was performed for 4h in a vacuum drying oven at 250 ^o^C. The U-shaped sample tube was used to load the powder for test. The adsorbed gas flowed through the sample in liquid nitrogen environment.

FTIR: The test wavelength ranges from 2000 to 400 cm^-1^.

XRD: Using a Cu-Ka1 radiation tube (voltage of 40 KV, tube current of 100 mA), the samples were scanned. The diffraction angle 2θ was selected for 10~70º with the scanning rate of 10º/min.

4. The porous structure in SEM image of Figure 1 is not clearly visible. Author are requested to provide a clear image.

Response：Thank you for pointing out. Due to the poor electrical conductivity of CuBG, it is difficult to obtain very clear images at a high magnification. We have added the full magnification image to the supplementary data as you suggested (Supplementary Fig.1).

5. Author should mention the wavelength of UV light used for photo crosslinking.

Response：Thank you for your suggestion! The wavelength of UV light for photo crosslinking is 365 nm. We have added this detail in the manuscript (line 186).

6. Please check the arrangement of sentences in section 2.7

Response：Great advice! We have rechecked the manuscript and rearranged the sentences in section 2.7.

7. Typo error in section 2.10 line 27, 2.11 line 47, 2.9 line 21, table 1

Response：Sorry for the mistakes! We have rechecked the manuscript carefully and correct the typo error which you mentioned.

8. Author has mentioned cytotoxicity analysis in the section 2.10 for BMSCs and hUVECs. The cell viability graphs for both cell lines should be provided in main article.

Response：Thank you for your advice! Due to the error in author's labeling, the CCK-8results and cell viability graphs of hUVECs have already been presented in the Supplementary Fig.3 & 4. Sorry for the mistake!

9. Caption of Figure 4 A should include the cells used for live/dead assay.

Response：Thank you for your suggestion! We have added the name of cells for live/dead assay as your request!

10. Author should maintain the consistency of using the word Figure/ Fig. in overall manuscript.

Response：Thank you for your suggestion! We have rechecked the article and unified the label of pictures as Fig. in overall manuscript.

11. Why in some experiments CB/PS is used whereas in some experiments BG/PS is used? Please explain.

Response：Great question! For most experiments, CB/PS was used and the CB here meant the BG doped with Cu. Only in the characterization of the angiogenic effect of Cu *in vitro* and *in vivo*, we used BG/PS to name the hydrogel containing the BG without Cu for contrastive study.

12. Mention Table 2 in main text.

Response：Thank you for your reminder! Table 2 showed the contents of Si, Ca, P and Cu in different groups of CuBG, which was detected by EDS. We have rechecked the manuscript and mentioned Table 2 in main text (line 299-301)!

13. Caption of supplementary figure 3. Please include the name of cells for live dead assay.

Response：Thank you for your advice! Supplementary figure 3 is a live/dead staining of hUVECs. We have added the name of cells in the caption of supplementary Figure 3.

14. The whole manuscript does not show the viability of hUVEC cells. Author should take care of this mistake. Authors should provide a detail scheme for better understanding of synthesis of final product.

Response：Sorry for the mistake! Due to the labeling error, Supplementary figure 2 & 3 should be CCK-8 cytotoxicity test and live/dead staining images for hUVECs. We have corrected this in the caption of figures. In addition, the schematic diagram of the synthesis of HAMA-PBA and the composite hydrogel process has been shown in Fig.1G and Fig.2A, respectively. HAMA-PBA was synthesized by an esterification and an amidation reaction. By one-pot method, CuBG and PS were mixed in PBS solution of HAMA-PBA and fully stirred to form the final product hydrogel. The specific gelation mechanism of hydrogel has also been shown in GA. The text has been slightly modified for better clarity (lines 333-334, 342-343).

15. What is DMTMM in section 2.5? Author should recheck the materials section and add this chemical in the section.

Response：Thank you for your question! DMTMM (4 -(4, 6-dimethoxy-1,3, 5-Triazin-2-yl)-4-methylmorpholinium chloride) is a carboxyl group activator which was used to activate the carboxyl group on hyaluronic acid for subsequent PBA grafting. We have rechecked the materials section add it into the section as you requested (lines 135-136).
Review Editor

Comments to the Author:

Please revise the graphic abstract.

Please notice that the GA should be prepared and displayed at the actual size of 1.4 inches (height) × 3.3 inches (width) or 3.6 cm × 8.4 cm following the Abstract in the submitted manuscript file.

Minimum resolutions are 300 d.p.i. for color or tone images, and 600 d.p.i. for line drawings. Preferred font type of the texts in the GA is Arial 8-16 points (smaller will not be legible). Try not to use the distracting and cluttering elements as much as possible. Please do not enlarge the GA, because the image will eventually appear on the first page of the published PDF file in the specified size above. For examples of graphical abstracts, please refer to the published papers since 2022.

Response：Thank you for your advice! We have revised our GA and resized it as you request.
